# Leiomyoma of esophagus—A case report

**DOI:** 10.1016/j.ijscr.2020.09.142

**Published:** 2020-09-23

**Authors:** Gowtham Thakut, Sheetal A. Murchite, Rajendra M. Kulkarni, Vaishali V. Gaikwad

**Affiliations:** Department of General Surgery, D. Y. Patil Medical College, Kolhapur, Maharashtra, 416006, India

**Keywords:** Esophagectomy, Gastrointestinal stromal tumors, Leiomyoma

## Abstract

**Introduction:**

Benign tumors of the esophagus are rare. However, among them, leiomyomas are common. Tumors larger than 5 cm are rare and may present as dysphagia or epigastric pain. Combined esophagoscopy and video-assisted thoracoscopic surgery or laparoscopic trans hiatal resection are used the management of leiomyoma of esophagus.

**Presentation of case:**

This case report illustrates the case of a young male who presented with dysphagia and occasional dyspnea. Imageology demonstrated an esophageal mass which on surgical exploration and histopathology, confirmed the diagnosis of leiomyoma.

**Discussion:**

Esophageal leiomyomas vary in their presenting size from a few centimeters to greater than 5 cm which are rare. The preferred surgical technique for leiomyomas is transthoracic enucleation without opening the mucosa, which is easier, faster, and safer compared to resection. In our patient, due to the size and location of the tumor, it was resected using an abdominal approach, and a distal esophagectomy and esophagogastrostomy were performed.

**Conclusion:**

A multi-modal approach involving both the endoscopic and radiologic examinations is crucial in ensuring that the morbidity of malignancy is avoided.

## Highlights

• Among the oesophageal tumour, only less than 10% are benign and leiomyoma is the most common tumour representing approximately two-thirds of the cases.
• Malignant transformation in leiomyomas are rare and cannot be accurately identified with needle aspiration biopsy making resection followed by histopathological evaluation vital.
• Malignant transformation in leiomyomas is rare and cannot be accurately identified with needle aspiration biopsy making resection followed by histopathological evaluation vital.

## 1 Introduction

Less than 10% of esophageal tumors are benign, among which leiomyoma is the most common tumor representing approximately two-thirds of the cases [1]. Esophageal leiomyomas are commonly found in middle-aged male patients with a male to female ratio of 2:1. Esophageal leiomyomas vary in their presenting size from a few centimeters to greater than 5 cm which are rare [2]. The larger tumors exhibit their presence as obstruction of the esophagus, symptoms due to tumor compression and/or dysfunction of the cardia but a great many of them are asymptomatic and are incidentally found [3]. Malignant transformation in leiomyomas is rare and cannot be accurately identified with needle aspiration biopsy making resection followed by histopathological evaluation vital. Transthoracic extra mucosal blunt enucleation is considered the standard surgical treatment. However, it may not be suitable for giant leiomyoma due to the mucosal damage and potential sarcomatous change of the tumor [4]. This project has been reported in line with the SCARE criteria [5]. Here we present a case of leiomyoma of the distal esophagus gastroesophageal junction and proximal stomach with concomitant dyspeptic symptoms.

## 2 Presentation of case

**Fig. 1:**
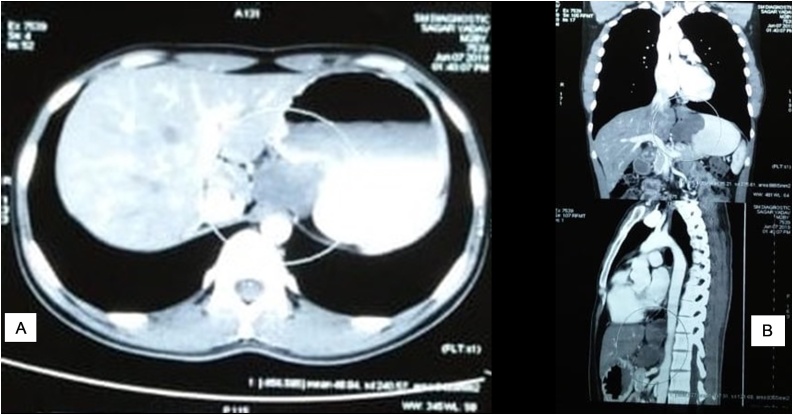
(A&B): CT scan showing the oesophageal lesion.

A 28-year-old male patient presented with a complaint of dyspepsia and esophageal reflux. A history of Nausea, vomiting, and weight loss were absent with no abnormality detected on physical examination and laboratory tests. Roentgenography of the chest revealed a mass with a filling defect in the right hemothorax Computerized tomography (CT) of the chest and abdomen showed a well-defined lobulated soft tissue mass (7 × 3.5 × 5.5 cm) on the posterior wall of the distal esophagus, gastroesophageal junction and adjoining portion of the posterior wall of the gastric fundus (Fig. 1).

**Fig. 2:**
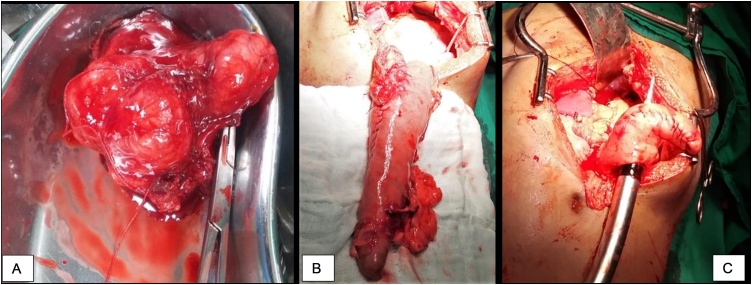
A) Showing Tumour B) Showing Gastric tube C) Showing Anastomosis with circular stapler.

Endoscopic examination of the upper gastrointestinal system showed a smooth and sessile submucosal growth at the fundus of the stomach. Transthoracic CT-guided Tru-cut biopsy followed by histopathological examination were performed which confirmed the diagnosis of leiomyoma of the esophagus with no malignant changes. A midline laparotomy was performed for the resection of the mass using distal esophagostomy, esophagogastrostomy and circular stapler. The excised mass was 7 × 6 × 2.5 cm in size and weighing 550gm (Fig. 2).

**Fig. 3:**
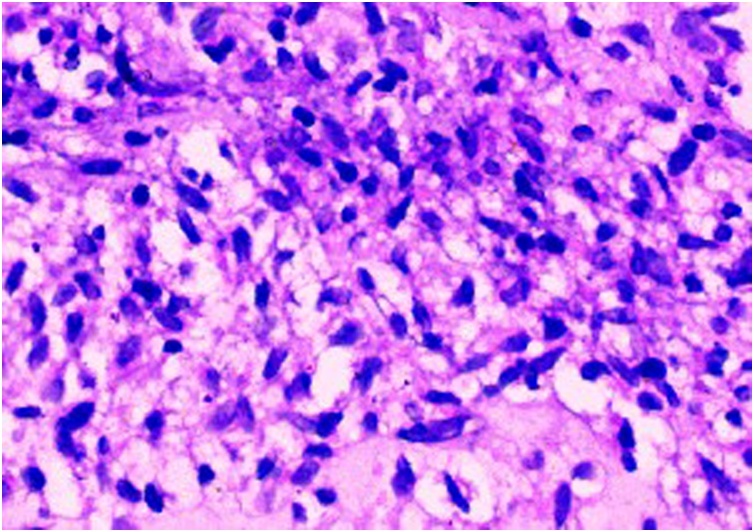
Showing Spindle cells (Microscopic).

Histopathology revealed the mass to be composed of neoplastic spindles cells arranged in bundles and fascicles. The cells had oval to elongated nuclei and a moderate amount of pale to eosinophilic cytoplasm, with mitotic figures less than 2 per 10 high power field and no evidence of necrosis (Fig. 3). A negative C kit was observed in immunohistochemistry study. The patient was advised to remain under follow up for 5 years.

## 3 Discussion

Leiomyoma often have an intramural location but some present near the esophageal diverticula or grow intramurally as a pedunculated polyp. Preoperative diagnosis of esophageal leiomyoma is often challenging as it can present as a posterior mediastinal mass on chest radiograph and may be seen as an incidental radiological finding [5]. Barium swallow is the commonly used imaging technique for the diagnosis of esophageal lesions by highlighting smooth filling defects in the esophageal lumen without mucosal damage. The tumor is usually mobile on deglutition [6,7]. CT and endoscopic ultrasound (EUS) play an important role in diagnosis as they delineate the intramural nature of tumors [8]. In this case, chest radiograph and CT scan revealed a well-defined soft tissue mass on the posterior wall of the distal esophagus, gastroesophageal junction. Use of biopsy in the diagnosis is controversial as debated in previous reports; it is associated with complications such as infection, bleeding, increased intraoperative perforation and technical difficulties [9,10]. Literature however also recommends that biopsy can be performed in case of a diagnostic dilemma [11]. CT-guided Tru cut biopsy was performed to confirm the diagnosis in this case.

The surgical indications of these tumors include unremitting symptoms, increased tumor size, mucosal ulceration, histopathologic diagnosis and facilitation of other surgical procedures [5]. Malignant transformation in leiomyomas is rare but needle aspiration biopsy usually does not accurately identify the nature of the lesion; therefore, malignancy can only be ruled out by resection [12]. Controversies do exist with respect to management as some recommend resection to rule out malignancy even when asymptomatic while others recommend a “follow up” policy [13,15]. Some recommended observation for asymptomatic patients with lesions smaller than 5 cm and when the preoperative workup has excluded malignancy [5]. The standard surgical approach is thoracotomy. The preferred surgical technique for leiomyomas is transthoracic enucleation without opening the mucosa, which is easier, faster, and safer compared to resection [5,13,14]. Esophageal resection is indicated for large tumors and tumors located at the gastroesophageal junction. Leiomyomas located at the proximal and middle third of the esophagus can be operated via a right thoracotomy [15,16]. Surgical resection is performed by a Trans Hiatal approach for tumors in the lower third of the esophagus. The video-assisted thoracoscopic approach with intraoperative esophagoscopy is another alternative that facilitates the procedure. EUS-guided endoscopic resection, endoscopic laser ablation, and aspiration lumpectomy are less invasive [13]. In our patient, due to the size and location of the tumor, it was resected using an abdominal approach, and a distal esophagectomy and esophagogastrostomy were performed.

## 4 Conclusion

The diagnosis of esophageal leiomyoma requires both endoscopic and radiologic examinations. Larger esophageal leiomyomas can be successfully resected using esophagectomy and esophagogastrostomy by abdominal approach. Stapler anastomosis can provide long survival and better quality of life. A multi-modal approach to ascertain diagnosis is crucial in ensuring that the morbidity of malignancy is avoided.

## Declaration of Competing Interest

Nil.

## Funding

Nil.

## Ethical approval

Ethical approval has been exempted by our institution.

## Consent

Written informed consent was obtained from the patient for publication of this case report and accompanying images. A copy of the written consent is available for review by the Editor-in-Chief of this journal on request.

## Author contribution

Case report concept and design was contributed by Dr. Sheetal A Murchite. Case report analysis was done by Dr. Rajendra M Kulkarni.

Data collection and case report writing was done by- Dr. Thakut Gowtham and Dr. Vaishali V Gaikwad.

## Registration of research studies

1. Name of the registry: NA.
2. Unique identifying number or registration ID: NA.
3. Hyperlink to your specific registration (must be publicly accessible and will be checked): NA.

## Guarantor

Dr. Thakut Gowtham.

Dr. Sheetal A Murchite.

Dr. Vaishali V Gaikwad.

## Provenance and peer review

Not commissioned, externally peer-reviewed.
